# Supplementary material for: How ATP and dATP Act as Molecular Switches to Regulate Enzymatic Activity in the Prototypical Bacterial Class Ia Ribonucleotide Reductase
Source: Biochemistry. 2024 Aug 20;63(19):2517–31. doi: 10.1021/acs.biochem.4c00329 (PMC11447812; doi:10.1021/acs.biochem.4c00329)
Supplement: Supplementary file 1 — bi4c00329_si_001.pdf [file bi4c00329_si_001.pdf]

Supplemental Tables and Figures

How ATP and dATP act as molecular switches to regulate enzymatic activity in the prototypical bacterial class Ia ribonucleotide reductase

Michael A. Funk[1, #], Christina M. Zimanyi[1, #], Gisele A. Andree[1], Allison E. Hamilos[1], and Catherine L. Drennan[1-4]*

AUTHOR ADDRESS

1 Department of Chemistry, Massachusetts Institute of Technology, Cambridge, MA 02139, USA

2 Department of Biology, Massachusetts Institute of Technology, Cambridge, MA 02139, USA

3 Howard Hughes Medical Institute, Massachusetts Institute of Technology, Cambridge, MA 02139, USA

4 Center for Environmental Health Sciences, Massachusetts Institute of Technology, Cambridge, MA 02139, USA

*Corresponding Author - Tel. (617) 253-5622, E-mail: cdrennan@mit.edu

#Authors contributed equally

Table S1. Primers used for mutagenesis experiments.

Primer	Sequence
α_2-β_{C35} fusion forward	GCCGTCAATCCAGGACGATGGCTCTGATAACGTGCAGGTTGC
α_2-β_{C35} fusion reverse complement	TACGCCGCATCCGGCATCTCAGAGCTGGAAGTTACTCAA
W28A mutagenesis forward	CGCGTTCTGGAT**GC**GGCGGCAGAAGGACTG
W28A mutagenesis reverse complement	CAGTCCTTCTGCCGCC**GC**ATCCAGAACGCG
F87A mutagenesis forward	CGCCTGGCGATC**GC**CCACCTGCG
F87A mutagenesis reverse complement	CGCAGGTGG**GC**GATCGCCAGGCG
F97A mutagenesis forward	GCCTACGGCCAG**GCG**GAGCCGCCTGCGCTG
F97A mutagenesis reverse complement	CAGCGCAGGCGGCTC**CGC**CTGGCCGTAGGC

Table S2. X-ray data collection statistics.

	α_2-dATP	α_2-(ATP)$_2$	α_2-β_{C35}-dATP
PDB ID	8VHO	8VHN	8VHU
Space group	$P4_32_12$	$P4_32_12$	$P2_12_12_1$
Cell dimensions (a, b, c; Å)	118.5, 118.5, 290.3	118.9, 118.9, 290.9	88.2 111.2 209.7
Wavelength (Å)	0.9793	0.9794	0.9792
Resolution (Å)	50.0-2.55 (2.59-2.55)	50.0-2.62 (2.67-2.62)	50-2.10 (2.14-2.10)
R_{sym}[a]	0.083 (0.830)	0.107 (0.772)	0.083 (0.801)
<I/σI>[a]	13.7 (2.0)	12.9 (2.7)	15.4 (2.7)
Completeness (%)[a]	99.9 (100.0)	99.4 (99.5)	98.7 (99.0)
Unique reflections	68422	63413	118973
Redundancy	4.8 (4.9)	7.2 (7.1)	8.9 (7.9)
CC1/2	0.694	0.756	0.79

	W28A α_2-(ATP)$_2$/CDP	W28A α_2-(dATP/ATP)	W28A α_2-(dATP/GTP)
PDB ID	8VHP	8VHQ	8VHR
Space group	$C222_1$	$P2_12_12_1$	$P2_12_12_1$
Cell dimensions (a, b, c; Å)	288.7 316.6 158.9	112.7 139.9 300.8	113.5 141.0 301.8
Wavelength (Å)	0.9792	0.9792	0.9792
Resolution (Å)	50.0-2.60 (2.64-2.60)	50.0-3.40 (3.52-3.40)	50.0-3.55 (3.68-3.55)
R_{sym}[a]	0.128 (>1)	0.094 (0.792)	0.075 (0.938)
<I/σI>[a]	12.7 (1.2)	9.8 (1.8)	11.9 (1.6)
Completeness (%)[a]	99.8 (99.9)	98.8 (97.1)	95.9 (93.6)
Unique reflections	218946	65872	57221
Redundancy	7.2 (6.4)	3.8 (3.6)	4.2 (4.0)
CC1/2	0.54	0.54	0.65

[a]Values for data in the highest resolution bin are shown in ().

Table S3. Crystallographic model refinement statistics for wild-type α_2 and α_2-β_{C35} structures.

	α_2-dATP	α_2-(ATP)$_2$	α_2-β_{C35}-dATP
PDB ID	8VHO	8VHN	8VHU
Resolution (Å)	50.0-2.55 (2.58-2.55)	50.0-2.62 (2.66-2.62)	50.0-2.10 (2.12-2.10)
Number of reflections	68422	63413	118834
R_{work}	0.165	0.166	0.154
R_{free}	0.205	0.209	0.188
R_{free} set size	3376 (4.9 %)	3103 (4.9 %)	3712 (3.1 %)
Model components			
Protein chains	2	2	2
Mg^{2+} molecules	4	4	3
dATP molecules	4	--	3
ATP molecules	--	6	--
MES molecules	2	2	--
Glycerol molecules	2	2	2
Cl^- ions	--	--	6
Na^+ ions	--	--	1
Water molecules	676	758	1357
Average B factors (Å^2)			
Protein	51.2	47.1	47.6
Mg^{2+} molecules	62.6	45.6	41.9
dATP (activity site 1)	72.3	--	54.6
dATP (specificity site)	43.2	--	36.0
ATP (activity site 1)	--	62.4	--
ATP (activity site 2)	--	79.1	--
ATP (specificity site)	--	48.8	--
MES molecules	114.6	105.6	--
Glycerol molecules	68.5	63.1	55.2
Cl^- ions	--	--	47.9
Na^+ ions	--	--	44.4
Water	57.9	51.0	55.6
Coordinate error (Å)	0.27	0.27	0.21
R.M.S deviations			
Bond length (Å)	0.002	0.007	0.003
Bond angle (°)	0.660	0.853	0.729
Rotamer outliers (%)	0.16	0.16	0.48
Ramachandran plot (%)			
Favored	97.88	97.74	97.80
Additionally allowed	1.85	2.12	2.06
Disallowed	0.27	0.14	0.14

Table S4. Model refinement statistics for W28A α_2 structures.

	W28A α_2-(ATP)$_2$/ CDP	W28A α_2- (dATP/ATP)	W28A α_2- (dATP/GTP)
PDB ID	8VHP	8VHQ	8VHR
Resolution (Å)	50-2.60 (2.67-2.60)	50-3.40 (3.45-3.40)	50-3.55 (3.59-3.59)
Number of reflections	218879	65402	56607
R_{work}	0.189	0.200	0.208
R_{free}	0.210	0.228	0.245
R_{free} set size	2000 (0.9 %)	3272 (5.0 %)	5641 (10.0 %)
Model components			
Protein chains	8	4	4
Mg^{2+} molecules	16	8	8
dATP molecules	--	8	8
ATP molecules	24	8	--
CDP molecules	8	--	--
GTP molecules	--	--	4
SO_4 molecules	40	--	--
Water molecules	1224	--	--
Average B factors (Å^2)			
Protein	54.2	125.7	145.7
Mg^{2+} molecules	53.3	85.9	113
dATP (activity site 1)	--	114	167
dATP (specificity site)	--	80.6	121
ATP (activity site 1)	59.8	--	--
ATP (activity site 2)	56.8	125	--
ATP (specificity site)	47.2	--	--
GTP (activity site 2)	--	--	178
CDP (substrate)	44.8	--	--
SO_4	60.7	--	--
Water	51.1	--	--
Coordinate error (Å)	0.31	0.37	0.48
R.M.S deviations			
Bond length (Å)	0.004	0.002	0.002
Bond angle (°)	0.762	0.581	0.618
Rotamer outliers (%)	0.36	0.04	0.44
Ramachandran plot (%)			
Favored	97.91	96.68	97.74
Additionally allowed	1.95	2.74	2.02
Disallowed	0.14	0.58	0.24

Table S5. Structural similarity between the cone domain of selected *E. coli* class Ia RNR crystal forms. All atom RMSD for residues 6-99 is given in angstroms. Only a single α chain was used for each structure.

	nucleotide free α_2 (1R1R)	$\alpha_4\beta_4$-dADP/dATP/CDP (5CNS)	α_2-(ATP)$_2$	α_2-dATP	α_2-β_{C35}-dATP	W28A α_2-dATP	W28A α_2-(ATP)$_2$	W28A α_2-(GTP/dATP)	W28A α_2-(ATP/dATP)
nucleotide free α_2		1.8	0.8	0.9	0.9	1.7	1.3	0.8	0.8
$\alpha_4\beta_4$-dADP/dATP/CDP	1.8		1.8	1.7	1.7	0.8	1.0	1.8	1.8
α_2-(ATP)$_2$	0.8	1.8		0.2	0.5	1.7	1.1	0.2	0.2
α_2-dATP	0.9	1.7	0.2		0.5	1.7	1.1	0.3	0.3
α_2-β_{C35}-dATP	0.9	1.7	0.5	0.5		1.7	1.0	0.6	0.6
W28A α_2-dATP	1.7	0.8	1.7	1.7	1.7		1.3	1.7	1.6
W28A α_2-(ATP)$_2$	1.3	1.0	1.1	1.1	1.0	1.3		1.1	1.1
W28A α_2-(GTP/dATP)	0.8	1.8	0.2	0.3	0.6	1.7	1.1		0.1
W28A α_2-(ATP/dATP)	0.8	1.8	0.2	0.3	0.6	1.6	1.1	0.1	

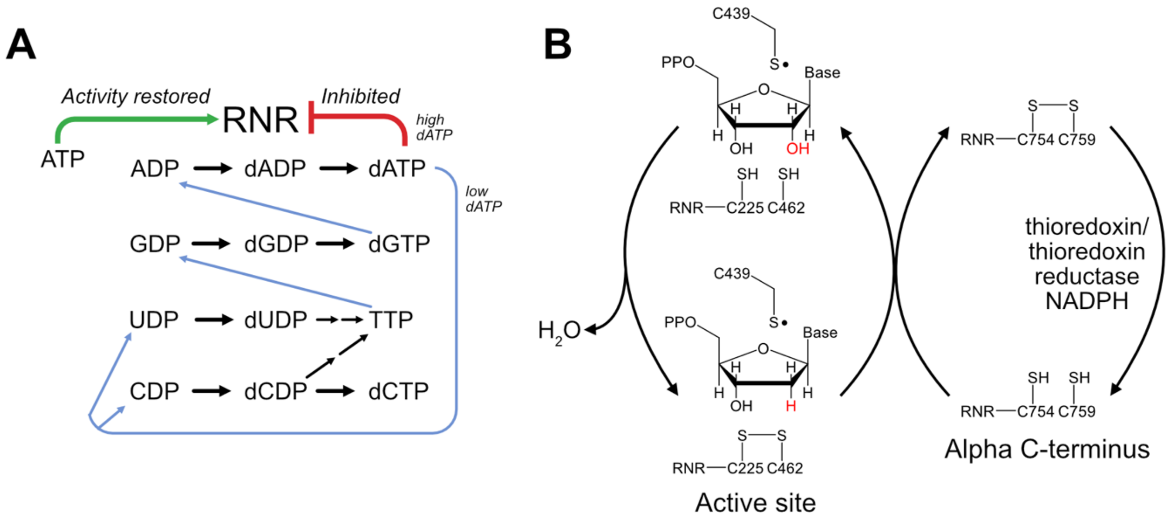

Figure S1. **Class Ia RNRs reduce all four ribonucleotide substrates and are allosterically regulated.** (A) Substrate specificity is regulated allosterically via the binding of deoxynucleotides to a specificity site at the dimer interface of α_2. dATP binding to the specificity site favors CDP and UDP reduction. TTP binding favors GDP reduction and dGTP binding favors ADP reduction. When the concentration of dATP increases, dATP binds to a second allosteric site, the activity site, inhibiting enzymatic activity. ATP can compete for binding at this activity site to restore activity. (B) Enzymatic turnover in class Ia RNR involves a series of redox-active cysteine pairs. The reducing equivalents for nucleotide reduction are initially provided by a pair of cysteines (C225/462) in the active site that are oxidized to form a disulfide concomitant with product formation. This disulfide is reduced by a second pair of redox-active cysteines (C754/759) found at the C-terminus of the α_2 subunit. Ultimately, the disulfide between C754 and C759 is reduced via the thioredoxin/thioredoxin reductase pair together with NADPH, thus allowing for additional rounds of turnover.

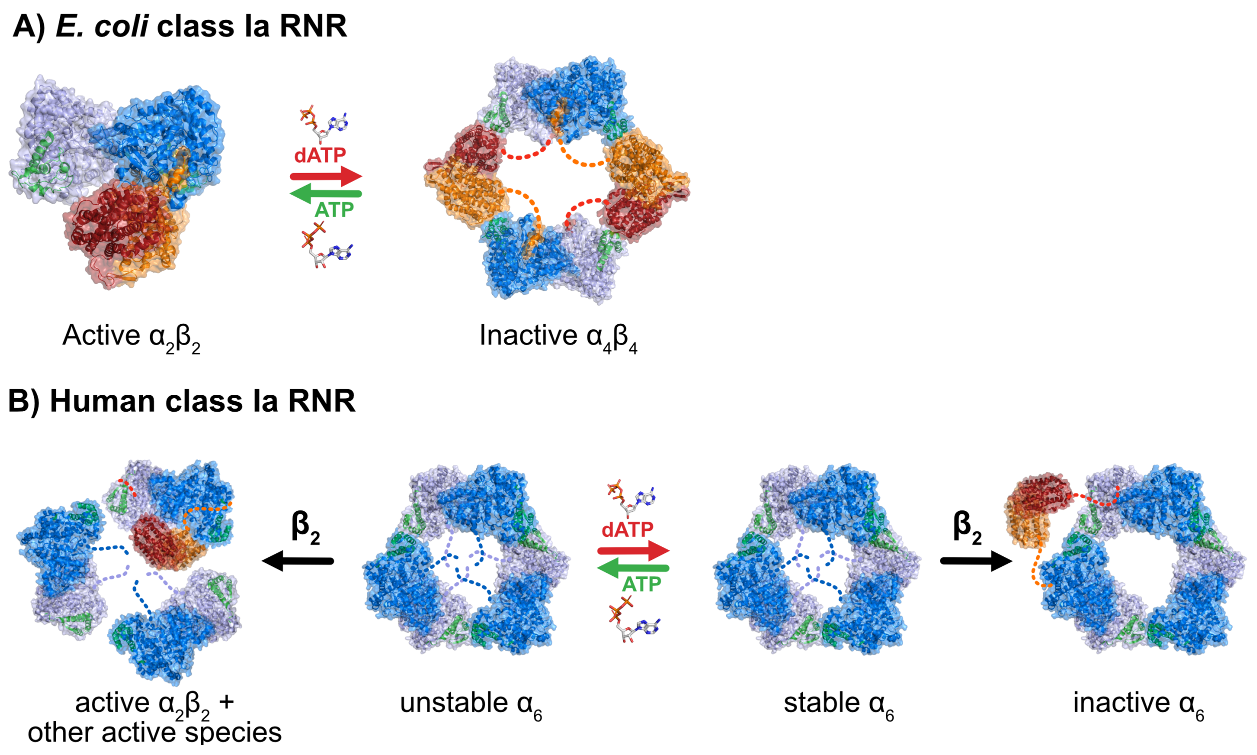

Figure S2. **Oligomeric changes in *E. coli* and human class Ia RNR**. (A) *E. coli* class Ia RNR transitions from an active complex (PDB: 6W4X) in the presence of ATP to an inactive $\alpha_4\beta_4$ ring-like structure (PDB: 5CNS) in the presence of dATP. (B) In the presence of ATP, human class Ia RNR forms an unstable α_6 complex, along with other species that include an active complex of α_2 and β_2 subunits. In the presence of dATP, human class Ia RNR forms an inactive, stable α_6 complex that is unable to position the β_2 subunit correctly for catalysis (α_6 PDB: 6AUI, β_2 PDB: 2UW2).

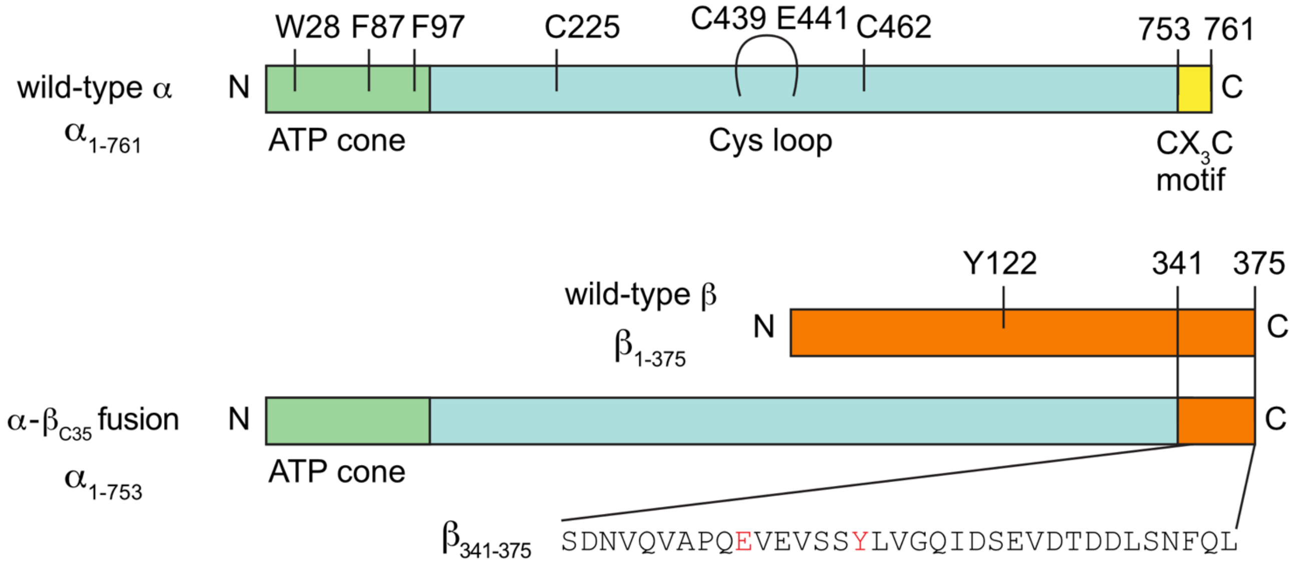

Figure S3. **Schematic diagram of protein constructs used in this study.** Residues chosen for substitution via site-directed mutagenesis within the cone domain are labeled as well as residues known to be involved in radical transfer or catalysis.

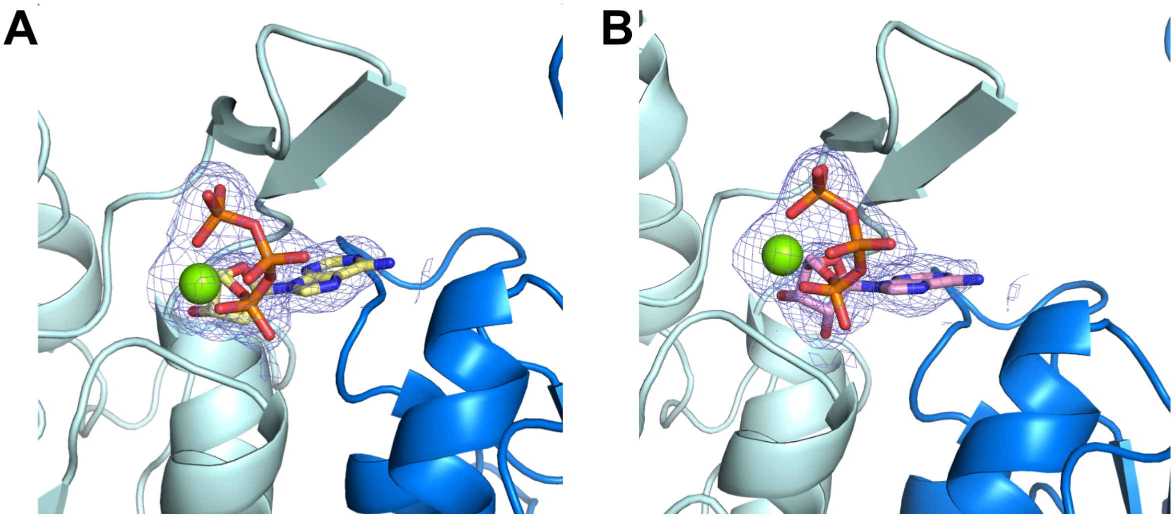

Figure S4. Omit electron density for ATP and dATP bound to the specificity site at the dimer interface of α_2 for *E. coli* class Ia RNR. (A) dATP (yellow carbons) bound at one of the specificity sites in wild-type α_2 dimer that also has dATP bound in the cone domain. (B) ATP (pink carbons) bound at one of the specificity sites in wild-type α_2 dimer that also has ATP bound in the cone domain. One monomer is shown in a darker shade of blue. Composite omit density contoured at 1.0 σ is shown in blue mesh.

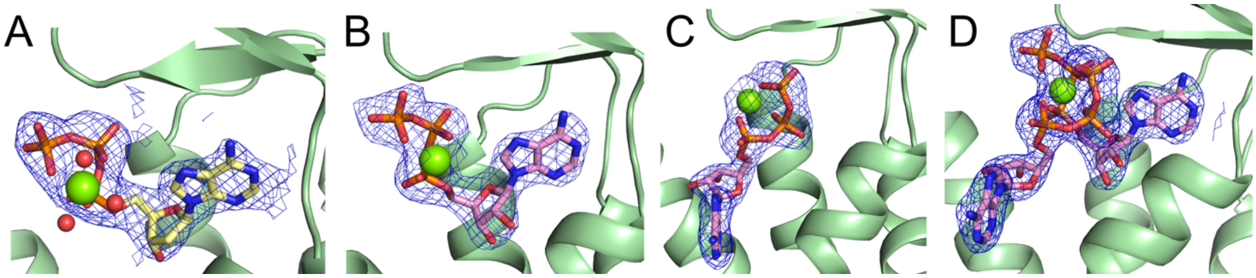

Figure S5. Omit electron density for ATP and dATP bound in activity site in the cone domain of α_2 for *E. coli* class Ia RNR. (A) dATP (yellow carbons) in site 1 of wild-type α_2-dATP. (B) ATP (pink carbons) in site 1 of wild-type α_2-(ATP)$_2$. (C) ATP in site 2 of wild-type α_2-(ATP)$_2$. (D) Both ATP molecules in the cone domain of wild-type α_2-(ATP)$_2$. Composite omit density contoured at 1.0 σ is shown in blue mesh. Site 1 and site 2 are explained in the main text.

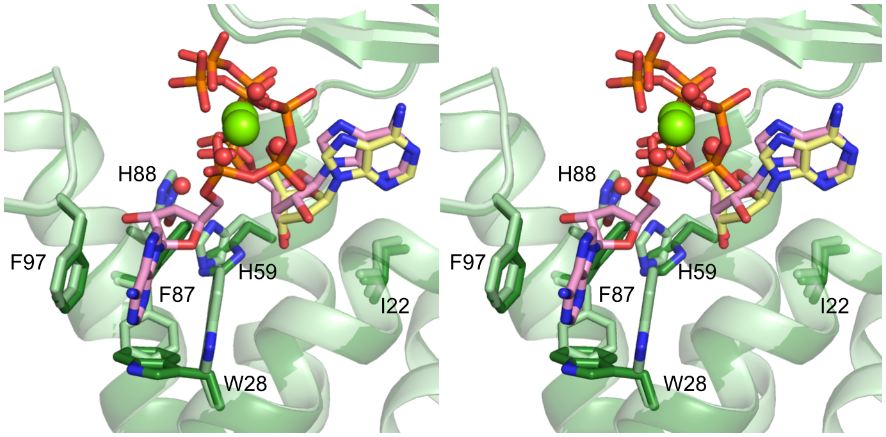

Figure S6. Stereoview of α_2-dATP structure superimposed with α_2-ATP structure. Stereoview of α_2-dATP (dark green, dATP carbons in yellow) aligned with α_2-$(ATP)_2$ (light green, ATP carbons in pink).

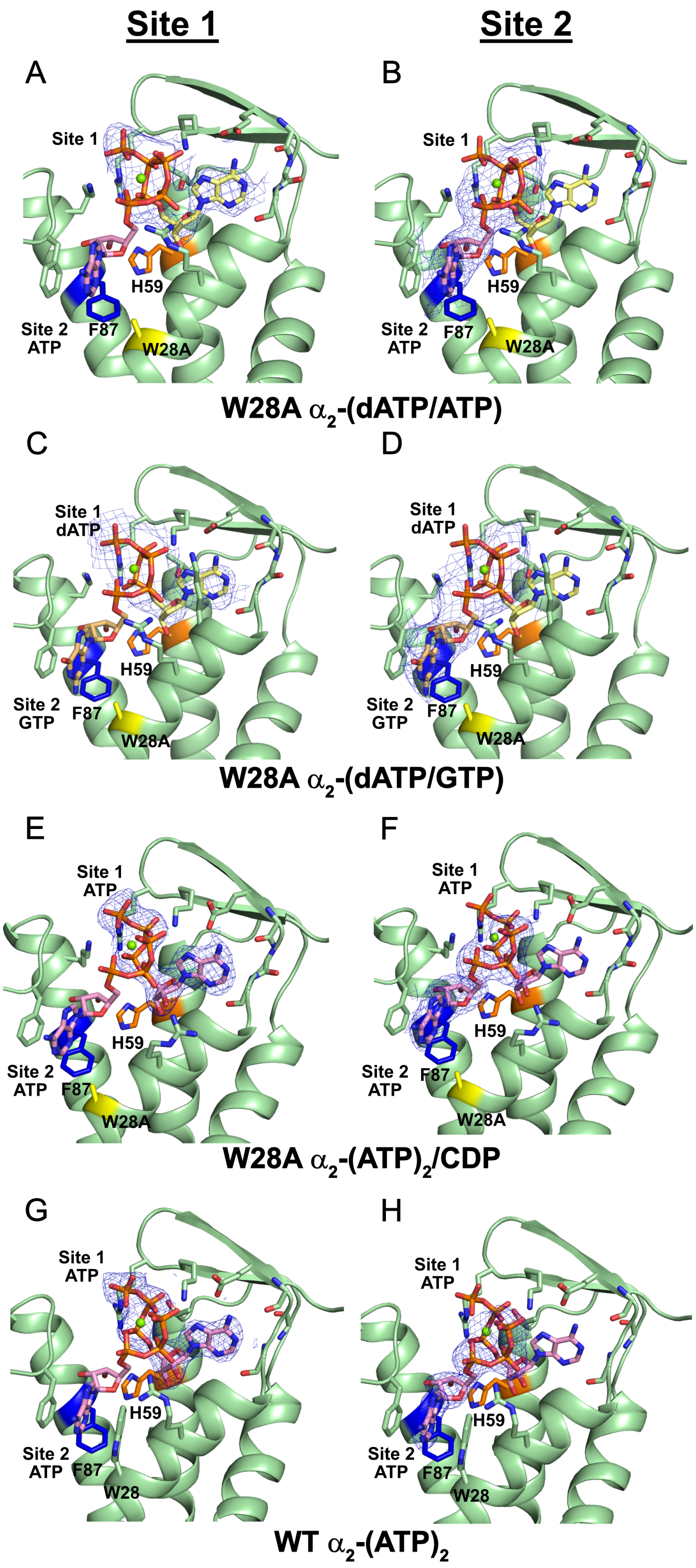

Figure S7. Omit electron density for activity effector binding in W28A structures compared to wild-type. (A) Omit map density for W28A α_2-(dATP/ATP) is not of sufficient quality to be able to distinguish ATP from dATP in site 1. Here we show dATP (yellow carbons) modeled in site 1. (B) Omit map for W28A α_2-(dATP/ATP) with ATP (pink carbons) modeled in site 2. (C) Omit map for W28A α_2-(dATP/GTP) with dATP (yellow carbons) modeled in site 1. (D) Omit map for W28A α_2-(dATP/GTP) with GTP (tan carbons) modeled in site 2. (E) Omit map for W28A α_2-(ATP)$_2$/CDP with ATP (pink carbons) modeled in site 1. (F) Omit map for W28A α_2-(ATP)$_2$/CDP with ATP (pink carbons) modeled in site 2. (G) Omit map for wild-type α_2-(ATP)$_2$ with ATP (pink carbons) modeled in site 1. (H) Omit map for wild-type α_2-(ATP)$_2$ with ATP (pink carbons) modeled in site 2_2. In all panels, F87 is in blue and H59 is in orange. Site of W28A substitution is highlighted in yellow in panels A-F. Composite omit density contoured at 1.0 σ is shown in blue mesh for one of the two nucleotides in each panel.

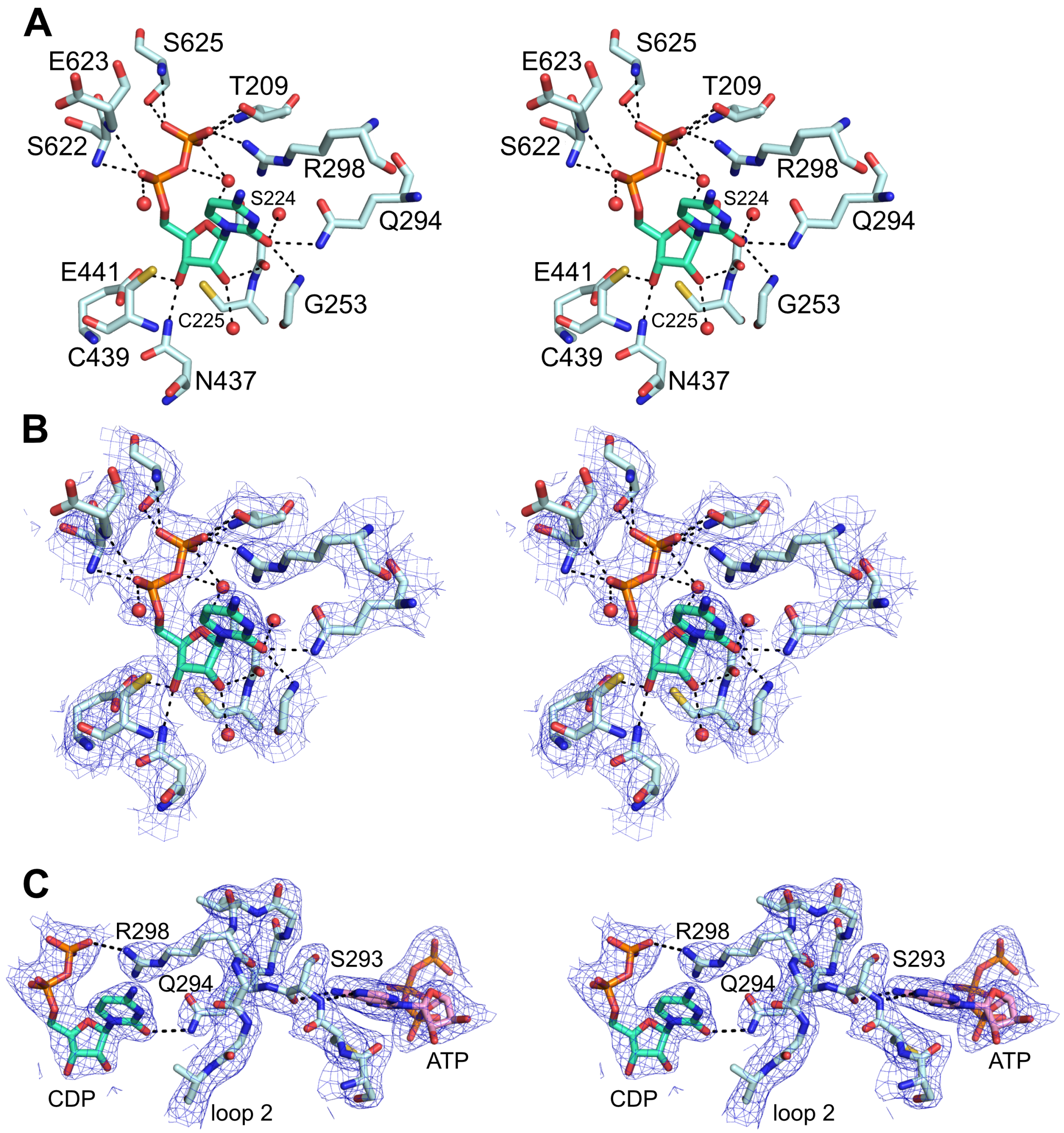

Figure S8. Stereoviews of CDP bound in the active site of W28A α_2-(ATP)$_2$ /CDP. (A) Wall-eyed stereoview of the interactions made by CDP (cyan carbons) with protein in the active site of W28A α_2-(ATP)$_2$/CDP. Hydrogen bonding interactions shown with black dashed lines. (B) Wall-eyed stereoview of composite omit electron density for CDP structure shown in A. Omit density contoured at 1.0 σ in blue mesh. (C) Wall-eyed stereoview of the interactions made by CDP (cyan carbons) and ATP (pink carbons) with the specificity loop (labelled loop 2). Hydrogen bonds (black dashed lines) between ATP and S293 backbone atoms of loop 2 orient the side chain of Q294 into the active site to hydrogen bond with the base of CDP. R298 from loop 2 contacts the CDP phosphates.

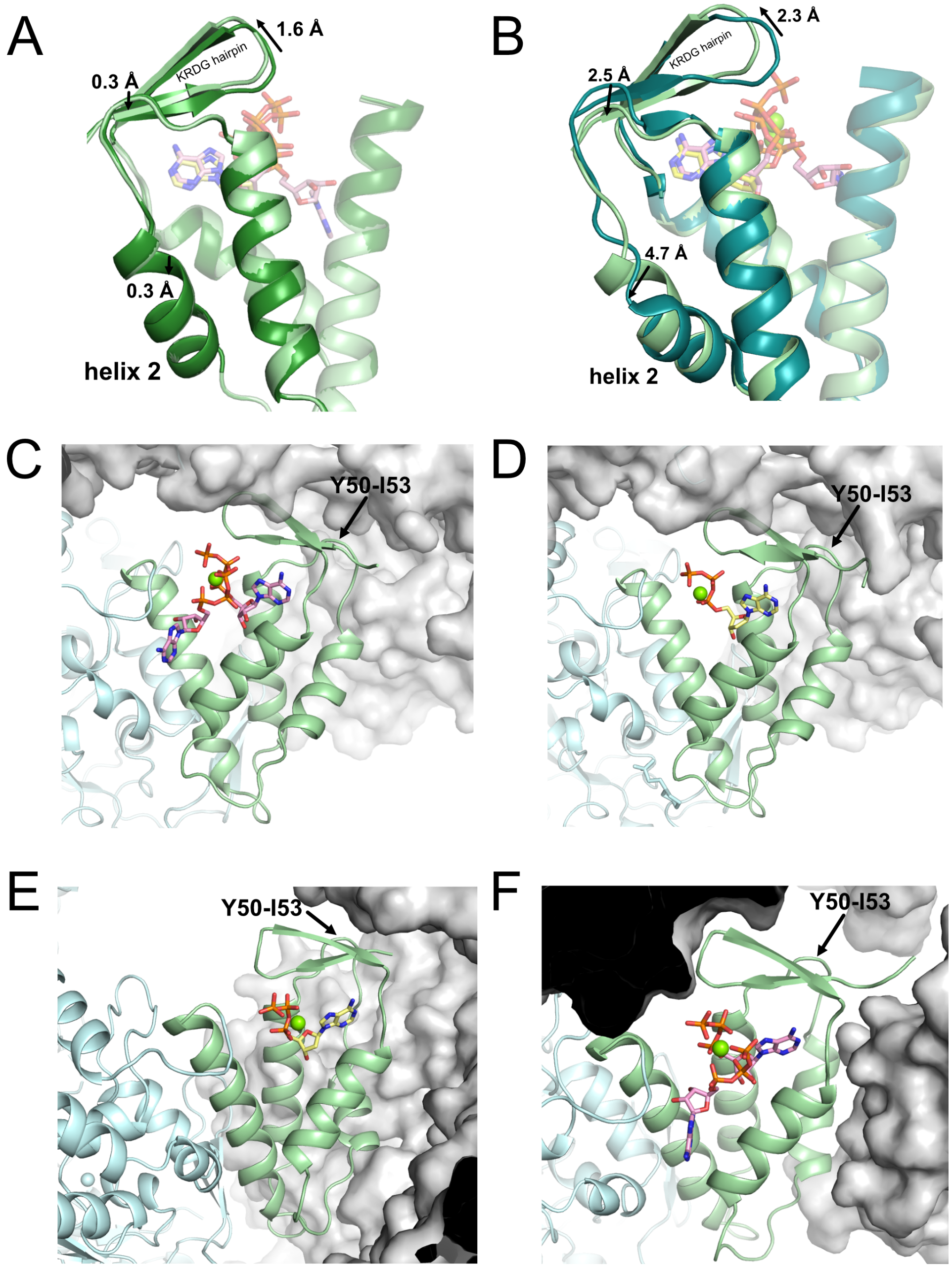

Figure S9. **Lattice contacts affect β-hairpin movement and thus the unwinding of helix 2 of the cone domain**. (A) Virtually no helix 2 unwinding is observed when α_2-$(ATP)_2$ (light green) and α_2-(dATP) (dark green) are compared. (B) Helix 2 unwinding seen in alignment of α_2-$(ATP)_2$ (light green) and $\alpha_4\beta_4$-(dATP) (PDB: 5CNS, dark teal). This panel is the same as Fig. 9B. (C-F) Lattice contacts of α2 structures shown with symmetry mates (grey surfaces). The cone domain is colored light green, the rest of protein is colored light blue, dATP carbons are shown in yellow and ATP carbons are shown in pink. Residues Y50-I53 that are at the base on the β-hairpin are highlighted with a black arrow. (C) α_2-$(ATP)_2$, (D) α_2-(dATP), (E) α_2-β_{C35}-(dATP) (F) W28A α_2-$(ATP)_2$

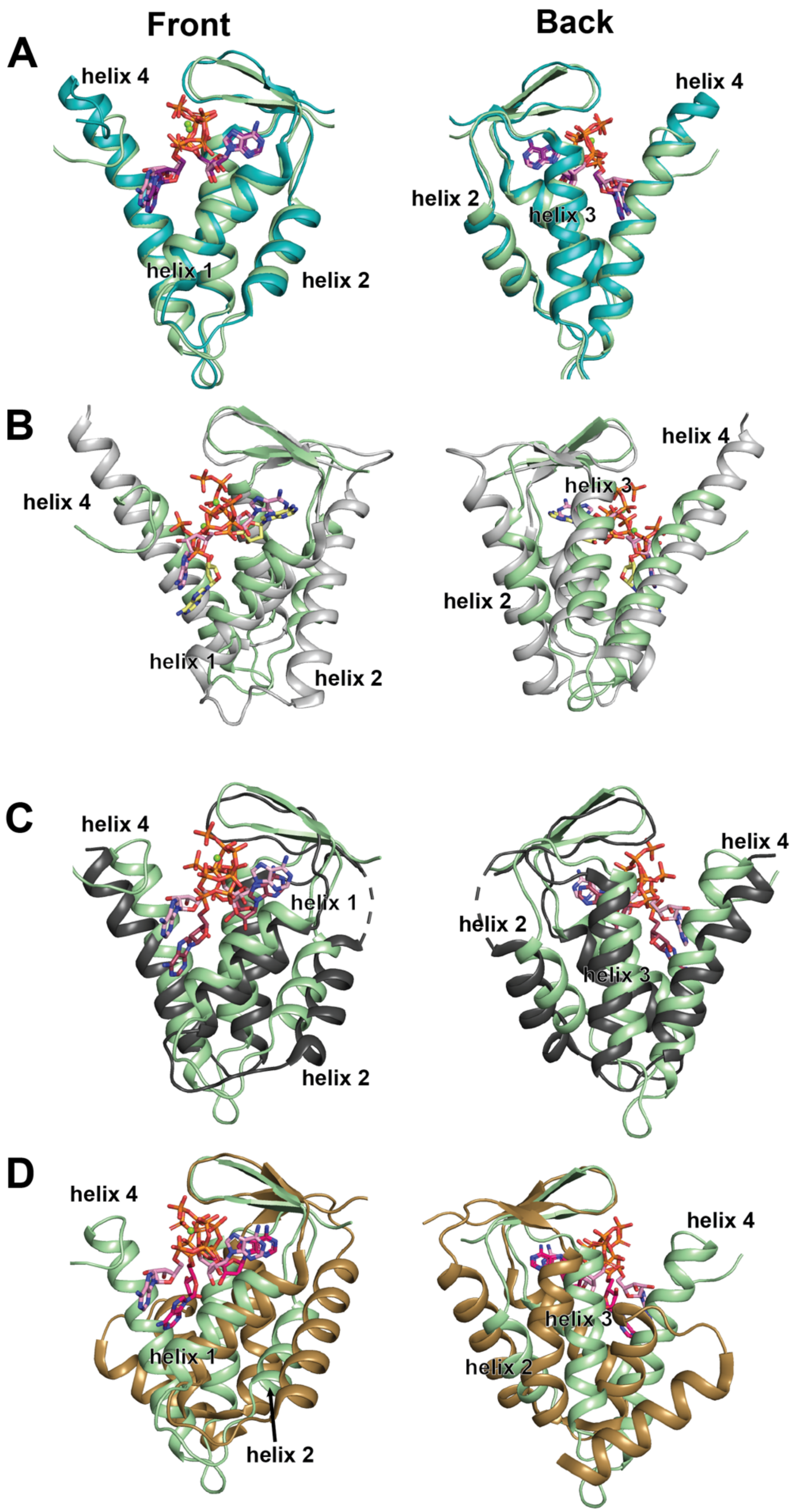

Figure S10. Structural comparisons of the α_2-(ATP)$_2$ *E. coli* class Ia cone domain with other cone domains that bind multiple nucleotides. For each alignment, the front view is shown on the left whereas the back view is shown on the right. Relevant helices are labeled. (A) α_2-(ATP)$_2$ *E. coli* class Ia cone domain (light green, ATP in pink carbons) superimposed with the (ATP)$_2$-bound *Aquiflex aeolicus* class Ia cone domain (teal, ATP in purple carbons). (B) α_2-(ATP)$_2$ *E. coli* class Ia cone domain (light green, ATP carbons in pink) superimposed with the (dATP)$_2$-bound *Pseudomonas aeruginosa* class Ia cone domain (light grey, dATP carbons in yellow). (C) α_2-(ATP)$_2$ *E. coli* class Ia cone domain (light green, ATP in pink carbons) superimposed with the (ATP)$_2$-bound *Prevotella copri* class III cone domain (dark grey, ATP in burgundy carbons). (D) α_2-(ATP)$_2$ *E. coli* class Ia cone domain (light green, ATP in pink carbons) superimposed with the (ATP)$_2$-bound *Streptomyces coelicolor* NrdR cone domain (brown, ATP in magenta carbons).

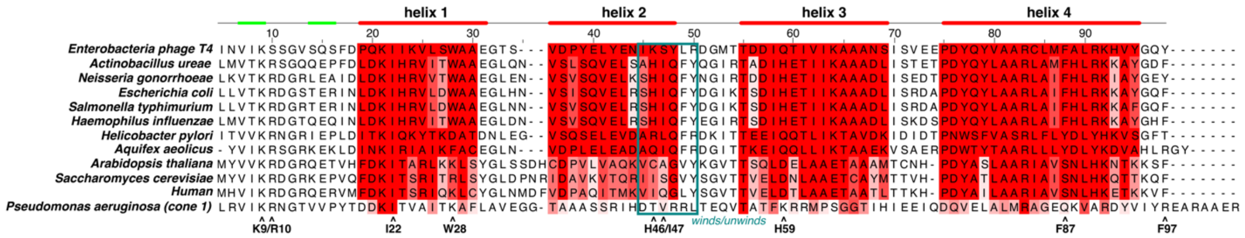

Figures S11. Alignment of selected class I RNR cone domain sequences. Cone domain sequences were obtained from UniProt and aligned using Clustal Omega. Sequences were chosen for alignment based on the availability of a three-dimensional structure and to represent the diversity of sequence space. Sequences are in order based on a neighbor joining tree using BLOSUM62 scoring matrix. Aligned sequences are colored by percent similarity to the *E. coli* class Ia cone domain helices (increasing red color indicates higher percent similarity). Residues are numbered according to the *E. coli* class Ia RNR residue numbers. Secondary structure for *E. coli* class Ia RNR is indicated at top of alignment (β-hairpin is indicted by green lines and helices by red lines). Residues discussed in this work are labeled on bottom of alignment.
